# Toxicity study in a pig model of intraperitoneal collagenase as an “enzymatic scalpel” directed to break stroma in order to generate a new perspective for peritoneal carcinomatosis approach: an experimental research

**DOI:** 10.1186/s12957-022-02524-2

**Published:** 2022-02-25

**Authors:** M. Garcia-Arranz, P. Villarejo-Campos, J. Barambio, S. Garcia Gomez-Heras, L. Vega-Clemente, H. Guadalajara, D. García-Olmo

**Affiliations:** 1 New Therapies Laboratory, Health Research Institute-Fundación Jiménez Díaz University Hospital (IIS-FJD), Avda. Reyes Católicos, 2, 28040 Madrid, Spain; 2 Department of Surgery, Universidad Autónoma de Madrid, C/ Arzobispo Morcillo s/n, 28034 Madrid, Spain; 3 Department of Surgery, Fundación Jiménez Díaz University Hospital, Avda. Reyes Católicos, 2, 28040 Madrid, Spain; 4 Department of Human Histology, Universidad Rey Juan Carlos, Avda de Atenas s/n, 28922 Alcorcón, Spain

## Abstract

**Background:**

This study aimed to measure the toxicity resulting from collagenase administration to the peritoneal cavity in a pig model as a preliminary step to break down the stroma surrounding tumors.

**Methods:**

Eight pigs were treated with 2 different collagenase concentrations previously tested in rats by our group. Time and temperature were controlled using a peritoneal lavage system (PRS System, Combat Medical Ltd.) identical to that used in human surgeries through hyperthermic intraperitoneal chemotherapy (HIPEC); 2 additional pigs were treated with peritoneal lavage only. Samples of blood and peritoneal fluid were collected pre-treatment, immediately after treatment, and 24 h postoperatively. In addition, histological studies and blood collagenase levels were measured.

**Results:**

No complications were observed during the surgeries. Intraoperative images evidenced the release of peritoneal tissue during collagenase treatment. After surgery, the animals showed no signs of pain. Diet and mobility were normal at 4 h postoperatively, and there were no significant differences in hematologic or biochemical parameters. Quantification of MMP1 and MMP2 in all samples as measured by absorbance showed no differences in blood collagenase levels between pre-treatment, post-treatment, and 24 h postoperatively.

None of the animals treated with collagenase showed peritoneal adhesions during the second surgery. Histologically, peritoneal organs and serous structures did not show any microscopic alterations associated with collagenase treatment in any group.

**Conclusion:**

Lavage of the peritoneal cavity with doses of up to 100,000 collagen digestion units/animal for 30 min is safe and removes connective tissue from the peritoneal cavity.

## Background

Solid tumors are surrounded by tumor stroma that is resistant to most drugs. This microenvironment protects the tumor and complicates nonsurgical approaches, which limits the ability of therapeutic agents to target cancer cells [1]. Disrupting this extracellular matrix, which is composed mainly of collagen, could facilitate the penetration of drugs into tumors and improve the therapeutic response [2–4]. Previous work by our group on rats has shown that intraperitoneal collagenase administered at a controlled time and concentration degrades the tumor stroma without damaging organs or other tissues [5]. In order to obtain authorization from regulatory agencies for peritoneal collagenase treatment in humans, we conducted a toxicity study in a large animal model.

Degradation of the extracellular matrix using intravenous collagenase has been shown to increase drug penetration in murine tumors [6]. Additionally, an approach using a system to apply collagenase in a rat tumor model has shown that proper control of enzyme time and concentration is essential [7].

In our previous study, we analyzed the effect of intraperitoneal collagenase on a rat model of peritoneal carcinomatosis. The present study is the first experiment performed in a large animal to demonstrate the non-toxicity of time- and concentration-controlled collagenase administration as a step prior to use in clinical practice. We decided to use the Landrace-White Langer pig since the coagulation parameters in adult animals are similar to those of humans [8]. The study was carried out in accordance with the recommendations of the Spanish Agency for Medicines and Health Products (AEMPS) as a prior step to authorizing a clinical trial in humans.

## Methods

Ten animals of both sexes, weighing between 25 and 30 kg, were divided into 3 groups. In group 1, 6 animals underwent lavage of the peritoneal cavity at 50,000 collagen digestion units (CDU) or 369.2 g of collagenase (GIDZyme-2 GMP, Spain) in 1.5 L of peritoneal dialysis solution; in group 2, 2 animals underwent lavage of the peritoneal cavity at 100,000 CDUs or 738.4 g of collagenase in 1.5 L of peritoneal dialysis solution, and in Group 3, lavage was performed in 2 animals with 1.5 L of the same solution. In all treatments, we used a temperature control system and peritoneal lavage (PRS System, Combat Medical, Ltd.). The procedure was approved by the Ethics Committee for Animal Welfare of the Health Research Institute-Fundación Jiménez Díaz and the regional government of Madrid (No.PROEX93.1/20). This report is in accordance with the ARRIVE guidelines (Animals in Research: Reporting In Vivo Experiments) [9]. The number of animals used was a recommendation of the OEBA to minimize the number of animals and generate pharmacological conclusions. All animals had an acclimation period of 48 h prior to surgery.

No prior randomization was performed. Trials were sequential, i.e., first a control group, followed by a second group with a low dose of collagenase treatment (50,000 CDU), and finally a group with the highest collagenase dose (100,000 CDU).

### Surgery

Following administration of anesthesia, animals were placed in the supine position, and a laparoscopic approach was performed using a Hasson trocar. Four 12-mm trocars were inserted and placed in the right upper quadrant, the left upper quadrant, the left iliac fossa, and the right iliac fossa. Subsequently, pressurization of the peritoneal cavity was performed using CO_2_ was, then the peritoneal cavity was tempered with washing solution preheated for at least 10 min. The next step was a peritoneal cavity lavage for 30min with collagenase (if applicable to the group) by means of the peritoneal lavage pump (Combat System). The filling volume of the abdominal cavity was individualized according to the capacity of each animal as determined using the PRS system (usually 1.5 L). A homogeneous intraperitoneal temperature of between 37 °C and 38 °C was maintained during all procedures. For intraoperative monitoring of hemodynamic parameters, we used the PiCCO® thermodilution system (PiCCO® catheter, Pulsion Medical Systems, Munich, Germany). Finally, the incision was closed with 2/0 vycril and the animal’s skin was sutured with 2/0 silk sutures. During surgery, to check the activity of the enzyme, a 0.5-cm incision was made in the abdomen through which an endoscopic camera was inserted to monitor the viability of the procedure and adjust washing times. All animals received adequate analgesia (tramadol 5 mg/kg weight) post-surgery for 24 h.

Twenty-four hours after treatment, the animals were operated under inhalational anesthesia by abdominal midline incision. Once the abdomen was examined and the samples were collected, the animals were euthanized with intravenous sodium thiopental.

In order to minimize possible confounding factors, all studies were carried out by the same team, only a researcher knew the applied treatment and the coding of the samples generated.

### Samples to collection

During surgery, physiological parameters were recorded (e.g., heart rate, PO_2_, temperature). Pre and post-treatment peritoneal lavage samples were collected to analyze collagenase levels.

In all cases, blood samples were obtained at 4 time points: after anesthesia, before starting surgery, immediately after the completion of peritoneal lavage, immediately after collagenase treatment (groups 2 and 3), and 24 h after peritoneal lavage. The following blood parameters were tested: ions, liver and pancreatic enzymes, blood count, and clotting. An external laboratory performed enzyme-linked immunosorbent assay (ELISA) tests against matrix metalloproteinase-1 (MMP1) and -2 (MMP2) according to the manufacturer's recommendations (Invitrogen). To measure statistical significance in MMP1 and MMP2 values, the Student’s *t* test was performed on the absorbance values distributed across the 3 groups using Microsoft Excel software.

Necropsy was performed 24 h after treatment; organs and tissues were macroscopically analyzed to detect damaged tissues, bleeding, and adhesions.

### Histological studies

We also carried out a histological examination of the samples of abdominal organs (i.e., spleen, liver, small intestine, kidney) and of the related serous membranes (peritoneum and pleura). Additionally, we analyzed the large intestine and abdominal wall for the presence of the same alteration by collagenase lavage.

After fixing the different samples in 4% formalin at room temperature, these were numerically coded and transferred to the Department of Human Histology of Rey Juan Carlos University (Madrid, Spain) for analysis. The fixed tissues were embedded in paraffin and cut into 5-micron-thick slices. Sections were stained with hematoxylin–eosin. All were studied under a Zeiss Axiophot 2 microscope and photographed with an AxiocamHRc camera.

## Results

No complications were observed during or after the surgeries: 4 h postoperatively, all animals drank water, displayed normal mobility, and showed no signs of pain. Intraoperative images obtained for animals in the groups receiving collagenase showed the release of peritoneal stroma during washing. No macroscopic changes were observed prior to treatment or 24 h postoperatively. There was no adhesion formation or bleeding in any case.

No laboratory, biochemistry, or hematologic values were observed to be ​outside the usual parameters in any case, any group, or at any time point (i.e., pre- and post-procedure and 24 h postoperatively). Fibrinogen was the only value found to be higher 24 h after treatment in all groups.

For collagenase levels in peritoneal lavage (post-lavage) and blood (pre-treatment, post-treatment, and 24 h post-treatment) all samples were coded and sent to the Living Cells company (Fuenlabrada, Spain).

The results of quantification of MMP1 and MMP2 measured by absorbance did not evidence differences in collagenase levels in blood or peritoneal lavage. For MMP1 measurements, 2 samples had slightly elevated levels of collagenase in the blood (0.019 to 0.056 and 0.62, respectively); these correspond to 1 pre-treatment and 1 post-treatment sample in 2 different animals of group 2 (Table 1). Regarding MMP2 data, all values were similar and high, except for the absence of statistically significant differences between samples (Table 1).

**Table 1:** ELISA for MMP1 and MMP2 in blood

Sample code	MMP1			MMP2		
	Sample	A 450/595 nm	ng/mL	Sample	A 450/595 nm	ng/mL
2	Pig 1 Pre	0.0165	24.84	Pig 1 Pre	2.012	74.54
5	Pig 1 Post	0.056	16.54	Pig 1 Post	1.448	47.62
7	Pig 1 24h	0.0155	25.44	Pig 1 24h	2.052	76.33
10	Pig 2 pre	0.014	25.29	Pig 2 pre	2.042	75.89
14	Pig 2 Post	0.0225	14.62	Pig 2 Post	1.318	43.88
18	Pig 2 24h	0.0195	26.57	Pig 2 24h	2.129	79.73
20	Pig 3 pre	0.018	18.74	Pig 3 pre	1.5975	56.23
17	Pig 3 post	0.02	23.28	Pig 3 post	1.905	69.84
9	Pig 3 24h	0.018	32.79	Pig 3 24h	2.551	98.38
6	Pig 4 pre	0.0195	18.19	Pig 4 pre	1.560	54.59
3	Pig 4 post	0.018	31.02	Pig 4 post	2.431	93.08
11	Pig 4 24h	0.062	26.07	Pig 4 24h	2.095	78.23
13	Pig 5 pre	0.0385	20.86	Pig 5 pre	1.741	62.59
19	Pig 5 post	0.0165	17.20	Pig 5 post	1.905	69.84
21	Pig 5 24h	0.021	25.46	Pig 5 24h	2.054	76.40
16	Pig 6 pre	0.033	14.93	Pig 6 pre	1.338	44.79
15	Pig 6 post	0.018	14.79	Pig 6 post	1.329	44.37
12	Pig 6 24h	0.018	29.39	Pig 6 24h	2.320	88.17
8	Pig 7 pre	0.0195	28.22	Pig 7 pre	2.241	84.66
4	Pig 7 post	0.0095	26.54	Pig 7 post	2.127	79.62
1	Pig 7 24h	0.0165	24.32	Pig 7 24h	1.976	72.97
24	Pig 8 pre	0.0155	25.44	Pig 8 pre	1.905	69.84
22	Pig 8 post	0.0146	25.32	Pig 8 post	2.040	75.88
23	Pig 8 24h	0.0145	25.32	Pig 8 24h	1.561	54.59

Absorbance values at 450 nm, taking the 595-nm reading as the noise value. The data is the mean of the duplicates. The extrapolated concentration of the MMP1 and MMP2 absorbance measures is included. Samples from pig 1–5 correspond to treatment group 1 (500,00 CDU collagenase), pig samples 6–7 correspond to treatment group 2 (100,000 CDU collagenase), and sample 8 corresponds to group 3 (control group)

**Fig. 1:**
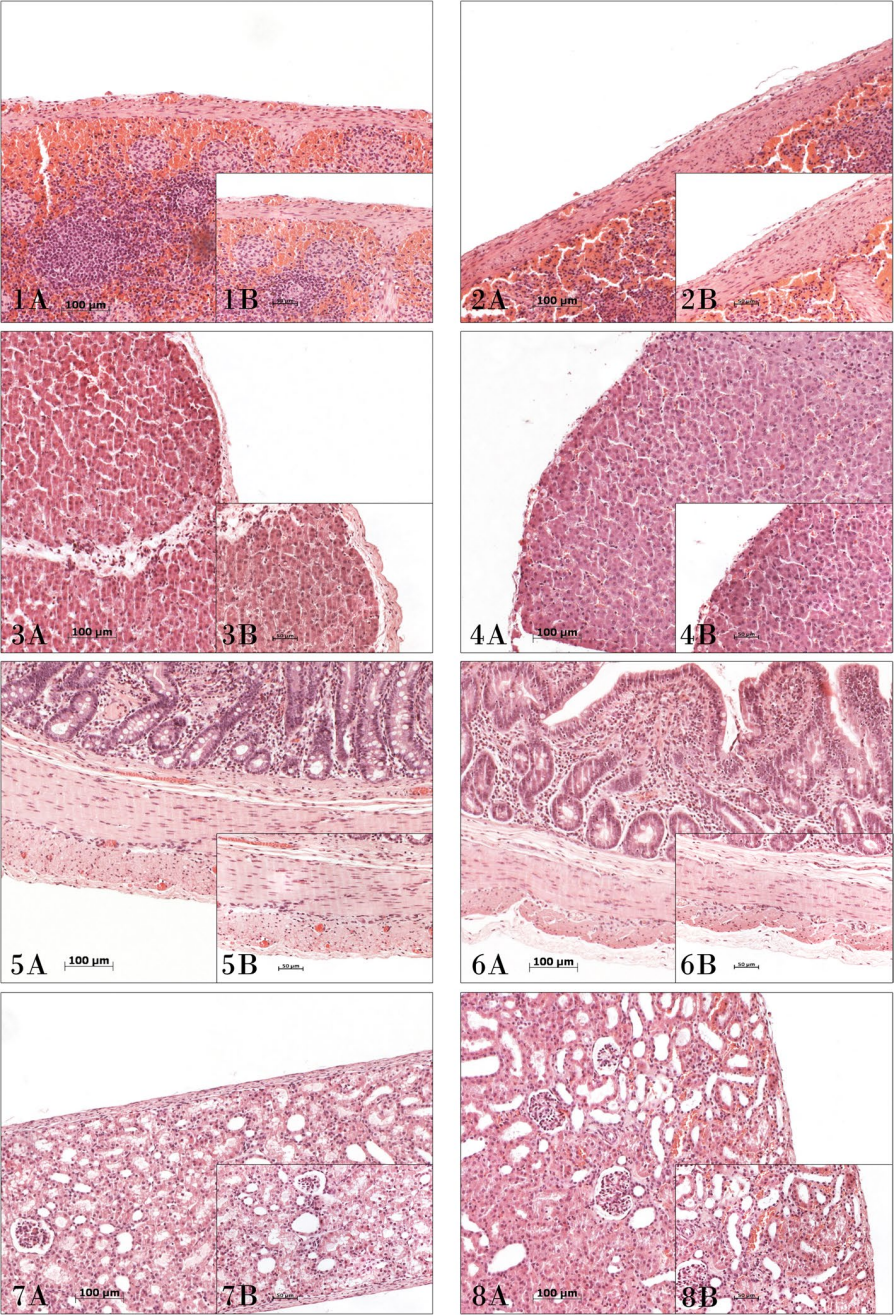
Histological study of principal peritoneal organs in treated groups. Histological images of the abdominal organs studied. Spleen (image **1**), liver (image **3**), small intestine (image **5**), and kidney (image **7**) of the group of animals treated with 50.000 CDU of collagenase. Spleen (image **2**), liver (image **4**), small intestine (image **6**), and kidney (image **8**) of the group of animals treated with 100,000 CDU of collagenase. Hematoxylin–eosin. (A) 100×. (B) 200×

The histology report found no altered structures in any abdominal organs in any group undergoing collagenase lavage, with particular focus placed on the concentrations in the spleen, liver, small intestine, and kidney by their connective tissue capsule (Fig. 1).

**Fig. 2:**
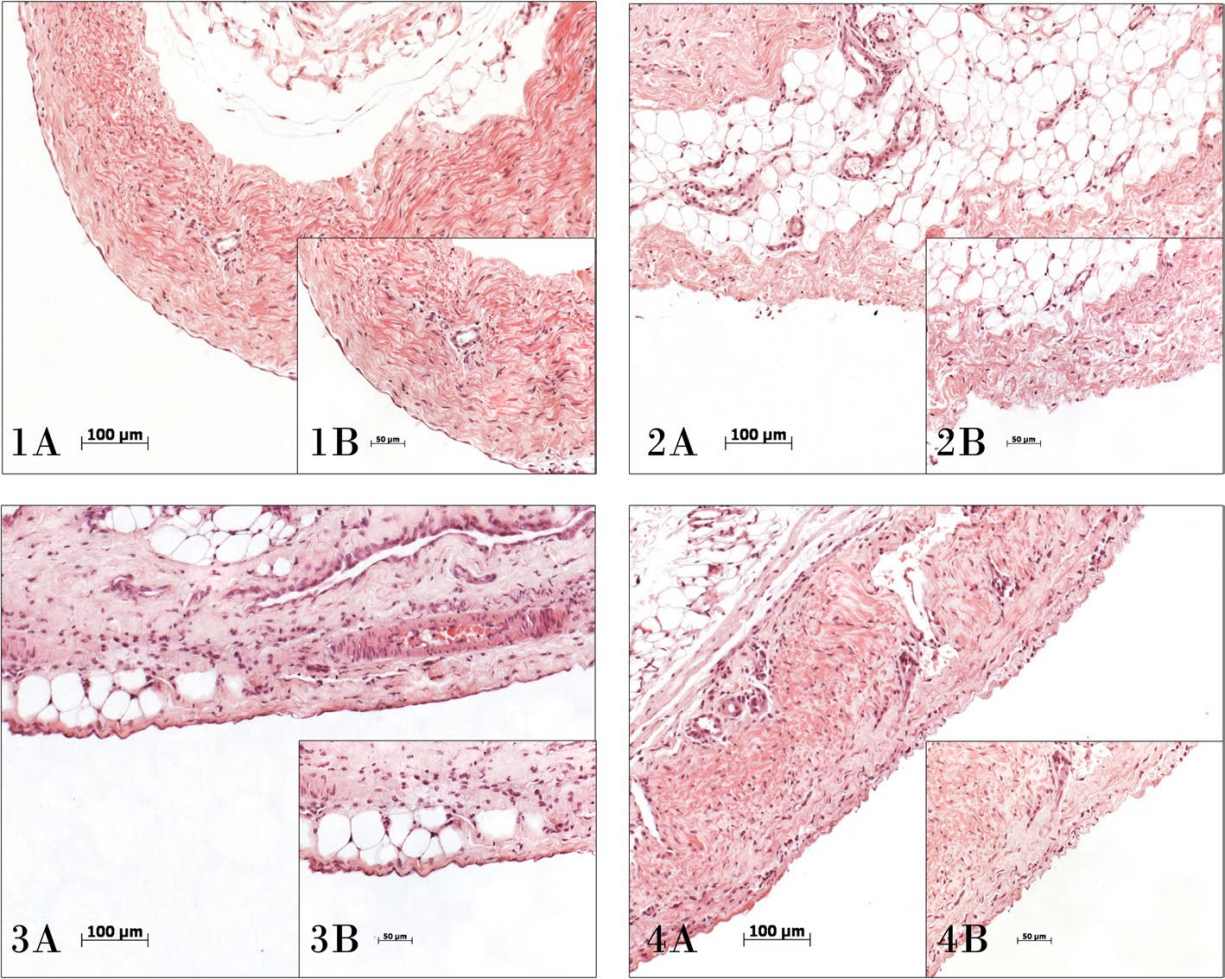
Histological study of serous membranes in treated groups. Histological images of the serous membranes studied. Peritoneum (image **1**) and pleura (image **3**) in the group of animals treated with 50,000 CDU of collagenase. Peritoneum (image **2**) and pleura (image **4**) of the group of animals treated with 100,000 CDU of collagenase. Hematoxylin–eosin. (A) 100×. (B) 200×

We also studied the serous membranes, peritoneum, and pleura. In none of them did we observe histologic alterations that could be caused by collagen at any concentration (Fig. 2).

## Discussion

Tumors characterized by an extracellular matrix with abundant collagen often have a poor response to drugs [10, 11]. The extracellular fibrous matrix of the tumor microenvironment acts as a barrier that hinders the delivery of the large-size molecules that make up the chemotherapy drugs used to target cancer cells, decreasing the tumor response [3]. These fibrous tumors include peritoneal cancers and metastases [12–14]. In recent years, it has been shown that collagenase treatment can limit fibrosis in liver tumors [15]. However, before they can be transferred to clinical practice, these in vitro studies and research on experimental models must demonstrate both efficacy and safety.

In our opinion, three aspects are essential to demonstrate safety: testing in a large mammal, histophysiologic monitoring of the organs that come into contact with collagenase, and measurement of collagenase in blood to determine any systemic effects. In our study, after controlling the collagenase concentration and the time of local application, we endeavored to analyze these aspects in a porcine model.

One important point is the period of activity of collagenase; some previous studies demonstrated that at 30 min collagenase activity is residual when injected into the peritoneal cavity of mice [4, 5]; in our assay and considering the high concentration of collagen existing in the peritoneal cavity, we are confident that that the catalytic activity of the enzyme has been exhausted at 30 min.

Our results show that is the animals are subjected to no post-treatment suffering despite administration of two doses of collagenase capable of releasing peritoneal stromal material. We observed no alterations in the biochemical or hematologic parameters in any of the 3 treatment groups, and only found constant elevation of the fibrinogen value at 4 and 24 h after surgery in all groups; we consider that this change may be associated with the surgical process.

Measurement of MMP1 values in blood to detect systemic collagenase activity was negative in all cases. The results for MMP2 did not reveal any differences between groups or as a function of sample collection time, although MMP2 was elevated in all cases. For MMP2, the report received from the laboratory that carried out the tests indicated that there had been a cross-reaction of the antibody with other porcine metalloproteases.

Finally, macroscopic and histologic study of the organs of the peritoneal cavity of all the animals has shown us that neither peritoneal lavage with saline solution nor the treatments performed with collagenase alters the organs at the proposed concentrations and with a lavage time of 30 min, this despite the finding of residual tissue and stromal cells in lavage.

Although we have focused on the toxicity of collagenase treatment by peritoneal lavage, we understand that our work has some limitations such as the non-generation of peritoneal carcinomatosis in a porcine model. Therefore, we have not analyzed the sequential effect of the proposed preconditioning followed by chemotherapeutic treatment, as we did in the murine model.

## Conclusions

We demonstrate that lavage of the peritoneal cavity for 30 min with 50,000 or 100,000 CDU of collagenase diluted in 1.5 L of saline solution between 36 and 38 °C is safe and does not generate any signs of toxicity in the treated animals.

Given the efficacy data previously obtained in a rat model [5], we propose in the future, a safety study to the health authorities through a clinical trial using collagenase lavage of the peritoneal cavity after completion of tumor cytoreduction to precondition the peritoneal surface in peritoneal solid tumors by HIPEC.

## Data Availability

The datasets analyzed during the current study available from the corresponding author on reasonable request

## Abbreviations

ARRIVE guidelines: Animals in Research: reporting In Vivo Experiments
CDU: Collagen digestion unit
ELISA: Enzyme-linked immunosorbent assay
HIPEC: Hyperthermic intraperitoneal chemotherapy
MMP 1: Matrix metalloproteinase I
MMP 2: Matrix metalloproteinase II
OEBA: Ethical Committee on Animal Welfare
